# A scanner system for high-resolution quantification of variation in root growth dynamics of *Brassica rapa* genotypes

**DOI:** 10.1093/jxb/eru048

**Published:** 2014-03-06

**Authors:** Michael O. Adu, Antoine Chatot, Lea Wiesel, Malcolm J. Bennett, Martin R. Broadley, Philip J. White, Lionel X. Dupuy

**Affiliations:** ^1^Department of Ecological Sciences, The James Hutton Institute, Invergowrie, Dundee, DD2 5DA, Scotland, UK; ^2^Plant and Crop Sciences Division, School of Biosciences, University of Nottingham, Sutton Bonington Campus, Leicestershire, LE12 5RD, UK

**Keywords:** Architecture, high-resolution, high throughput, model, nitrogen, phenotyping, phosphorus, root.

## Abstract

The potential exists to breed for root system architectures that optimize resource acquisition. However, this requires the ability to screen root system development quantitatively, with high resolution, in as natural an environment as possible, with high throughput. This paper describes the construction of a low-cost, high-resolution root phenotyping platform, requiring no sophisticated equipment and adaptable to most laboratory and glasshouse environments, and its application to quantify environmental and temporal variation in root traits between genotypes of *Brassica rapa* L. Plants were supplied with a complete nutrient solution through the wick of a germination paper. Images of root systems were acquired without manual intervention, over extended periods, using multiple scanners controlled by customized software. Mixed-effects models were used to describe the sources of variation in root traits contributing to root system architecture estimated from digital images. It was calculated that between one and 43 replicates would be required to detect a significant difference (95% CI 50% difference between traits). Broad-sense heritability was highest for shoot biomass traits (>0.60), intermediate (0.25–0.60) for the length and diameter of primary roots and lateral root branching density on the primary root, and lower (<0.25) for other root traits. Models demonstrate that root traits show temporal variations of various types. The phenotyping platform described here can be used to quantify environmental and temporal variation in traits contributing to root system architecture in *B. rapa* and can be extended to screen the large populations required for breeding for efficient resource acquisition.

## Introduction

Breeding crops with better root system architectures (RSAs) for the acquisition of water and mineral elements and, thereby, greater resource-use efficiency, requires the ability to screen root system development quantitatively, in high resolution, nondestructively, in as natural an environment as possible, on a large number of genotypes in a short time (de Dorlodot *et al.*, 2007; Walter *et al.*, 2007; Zhu *et al.*, 2011; Fiorani and Schurr, 2013). Traditional techniques used in the field include soil coring (Box and Ramsuer, 1993), trenching (Vepraskas and Hoyt, 1988), and pinboard excavation (Oliveira *et al.*, 2000), followed by washing substrate from the roots and quantification of root length and diameters. These techniques are slow and laborious, destructive, prone to inaccuracy (because small roots are lost during washing), and ill suited to screening large genetic populations (Smit *et al.*, 2000; Trachsel *et al.*, 2010).

To overcome some of the limitations of phenotyping root systems in the field, researchers have developed methods to phenotype the root systems of plants growing in artificial substrates under controlled conditions in the laboratory or glasshouse. Various translucent, artificial media have been employed, including water (Drew and Saker, 1975; Tuberosa *et al.*, 2002), aeroponics (Waisel, 2002; Eshel and Grunzweig, 2013), gels (Bengough *et al.*, 2004; Shi *et al.*, 2013), and transparent soils (Downie *et al.*, 2012). The use of transparent artificial substrates has many advantages. First, the homogeneity of the media is controlled, and therefore it is possible to minimize the inherent variability of the root traits observed. Imaging is facilitated in clear media, and the application of automated algorithms for the extraction of root features is, therefore, possible (French *et al.*, 2009). The light spectrum can be exploited to improve image quality and reduce the effects of high light doses on root growth (Yazdanbakhsh and Fisahn, 2009). Using dyes and fluorescence imaging, it is also possible to characterize functional traits, such as apoplastic pH (Bibikova *et al.*, 1998). Biospeckle laser imaging, a more recent technique, provides new opportunities to screen for functional traits without the use of a dye (Ribeiro *et al.*, 2014). Finally, transparent substrates allow 3D descriptions of RSAs using a range of techniques such as laser scanning (Fang *et al.*, 2009), optical (Iyer-Pascuzzi *et al.*, 2010; Clark *et al.*, 2011), or light sheet tomography (Yang *et al.*, 2013).

Although these techniques facilitate nondestructive analyses of RSAs, root traits of plants grown in these substrates are not always well correlated with those of plants grown in the field (Wojciechowski *et al.*, 2009; Schmidt *et al.*, 2012). Another common technique is to observe roots growing at the interface between soil and a transparent barrier. This includes observations from belowground rhizotrons (Bland *et al.*, 1990) or minirhizotrons inserted into the soil (Zeng *et al.*, 2008; Dupuy *et al.*, 2010) and observations of plants growing in rhizotubes or rhizoboxes (Nagel *et al.*, 2012; Dresbøll *et al.*, 2013). However, these techniques provide only partial information about RSAs and can affect plant growth by physical interactions (Wenzel *et al.*, 2001).

Recently, radiation-based techniques, such as nuclear magnetic resonance imaging (Rascher *et al.*, 2011) and neutron and X-ray computed tomography (Oswald *et al.*, 2008; Flavel *et al.*, 2012; Mairhofer *et al.*, 2013), have become popular because they allow noninvasive measurements of RSAs in soils. However, instrumentation costs are generally high and the acquisition of data is often too slow to enable dynamic measurements of root system development or the screening of large genetic populations (Iyer-Pascuzzi *et al.*, 2010; Smith and De Smet, 2012; Fiorani and Schurr, 2013).

This paper describes a low-cost, high-resolution root phenotyping platform that requires no sophisticated equipment and is adaptable to most laboratory and glasshouse environments. It is based on a traditional pouch-and-wick system (Liao *et al.*, 2001; Hund *et al.*, 2009) in which roots are grown on the surface of germination paper and imaged in high resolution using flatbed scanners. Images were acquired without manual intervention, over extended periods, using multiple scanners controlled by customized software. The platform was used to screen RSAs of up to 48 plants simultaneously and has the potential to be expanded. The platform was used to estimate the number of replicates required to detect differences in traits contributing to RSAs between genotypes of *Brassica rapa* L. and to quantify genotypic, environmental, and temporal variation in these traits.

## Materials and methods

### Genetic material

The variability of root architectural traits was studied in a diploid inbred line of *B. rapa* L. subsp. *trilocularis* cv. R-o-18 (Stephenson *et al.*, 2010). Two parents (cv. IMB211 and cv. R500) and 14 recombinant inbred lines (RILs) of the BraIRRI mapping population were used to study variations in root traits caused by genetic factors. The BraIRRI population is an immortal mapping population consisting of 160 RILs derived from the cross between IMB211 and R500 (Iniguez-Luy *et al.*, 2009). Genotype IMB211 is a highly inbred rapid cycling Chinese cabbage *B. rapa* subsp. *pekinensis* and R500 is a highly inbred annual yellow sarson *B. rapa* subsp. *trilocularis* (Iniguez-Luy *et al.*, 2009; Xu *et al.*, 2010).

### Growth conditions

**Fig. 1.**
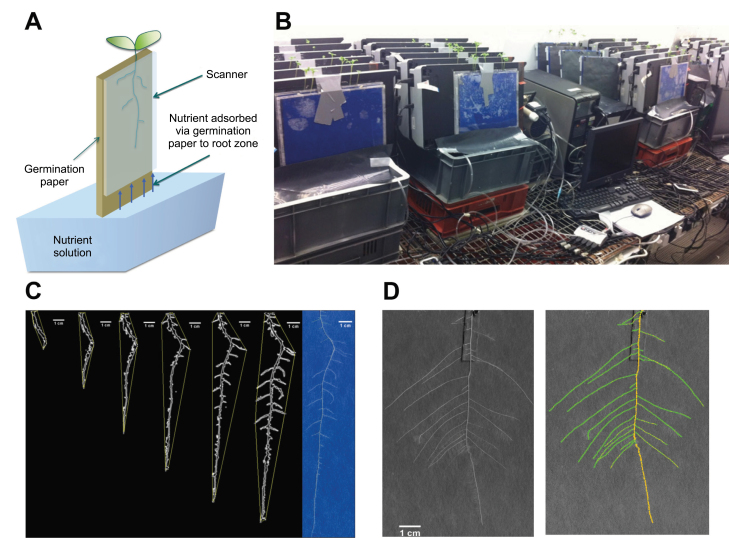
(A) Schematic representation of the pouch-and-wick system used to grow plants in the phenotyping platform. Roots grew on the surface of germination paper held between a clear-Perspex plate and the glass window of a scanner. Scanners were fixed in near-vertical positions 5cm above 20 l of nutrient solution contained in opaque polyvinyl plastic tanks each supplying six scanners. Approximately 10cm of the germination paper was submerged in the nutrient solution. (B) The phenotyping platform comprising 24 scanners assembled in four banks of six scanners. (C) The features of a root system, including the convex hull, at successive timepoints obtained using customized ImageJ macros, including the root system at the last timepoint. (D) The features of a root system at a specific timepoint obtained using the SmartRoot plugin for ImageJ.

Plants were grown using a pouch-and-wick system (Liao *et al.*, 2001; Hund *et al.*, 2009). Seeds were sown on 12×12cm germination papers (Anchor Paper, Saint Paul, MN, USA) sprayed with deionized water and placed vertically in a Sanyo MIR153 incubator at 20 °C. Three days after sowing (DAS), seedlings of similar size with radicles 2–3cm in length were transferred to large sheets of germination paper (30×42cm) attached to flatbed scanners using 30×20cm clear-Perspex plates (Fig. 1a). The germination paper surrounding each radicle was cut and transferred with the seedling to minimize disturbance during this process. Two seedlings were placed on each large sheet of germination paper. Scanners were fixed in near-vertical positions 5cm above 20 l of nutrient solution contained in opaque polyvinyl plastic tanks, each supplying six scanners (Fig. 1b). Approximately 10cm of the germination paper was submerged in the nutrient solution.

Nutrient solution was prepared with deionized water and contained 2mM Ca(NO_3_)_2_, 2mM NH_4_NO_3_, 0.75mM MgSO_4_, 0.5mM KOH, 0.25mM KH_2_PO_4_, 0.1mM FeNaEDTA, 30mM H_3_BO_3_, 25mM CaCl_2_, 10mM MnSO_4_, 3mM CuSO_4_, 1mM ZnSO_4_, and 0.5mM Na_2_MoO_4_ (Broadley *et al.*, 2003). The nutrient solution was adjusted to pH 6 at the start of the experiment using H_2_SO_4_. Plants were grown under a 16/8h day/night cycle. Temperature in the growth room was kept constant at 15 °C. Light intensity during the day was maintained at 100 μmol m^–2^ s^–1^ at plant height. Relative humidity was approximately 60%. Seedlings were removed from scanners at 18 DAS. Roots were excised from the shoot base and freshweight (FW) of roots and shoots was recorded. Shoot and root samples were dried at 60 °C for 72h and dry weight (DW) was determined. The single genotype experiment on R-o-18 was performed in five independent runs, each run comprising eight scanners with two plants per scanner. Trait data was collected on 72 plants. The multiple-genotype experiment on the 16 BraIRRI lines was performed in two independent runs, each run comprising two consecutive assays of a subset of eight genotypes grown in four banks of six scanners with two plants per scanner. Trait data was collected on 190 plants.

### Time-lapse imaging of roots

Images were taken daily from 3 to 18 DAS using flatbed scanners. Scanners were chosen for this purpose because their resolution is high compared to standard cameras. Thus, fine roots and root hairs have the potential to be resolved. In this study, 24 A4 CanoScan 5600F scanners (Canon UK, Reigate, UK) were assembled in four banks of six scanners (Fig. 1b). This allowed the root systems of 48 seedlings to be imaged simultaneously. The frequency of image acquisition, scanning resolution, and file format was controlled by three computers using in-house software (ArchiScan). ArchiScan was programmed in Python and employs the TWAIN module (http://twainmodule.sourceforge.net/) for communicating between computers and scanners. The program is distributed under the GPL2 open source license and can be downloaded from the ArchiRoot website (http://www.archiroot.org.uk). ArchiScan is generic software and can be used with any scanner provided a TWAIN driver is available.

### Image segmentation and extraction of root features

Features of a root system at specific timepoints were determined using ImageJ (http://rsbweb.nih.gov/ij/), using either in-house macros that can be downloaded from the ArchiRoot website (Fig. 1c) or the SmartRoot plugin (Fig. 1d; http://www.uclouvain.be/en-smartroot). Image analysis was carried out on greyscale images obtained from the red channel of the colour images. Median and Gaussian filters were applied to remove noise resulting from, for example, condensation on the surface of scanners or differences in the texture of the germination paper. Variations in pixel intensity over longer distances than the root diameter resulting from, for example, nonuniform moisture content of the germination paper were removed by subtracting the mean background pixel intensity of neighbouring pixels from the pixel intensity of the original image. Macros implemented: (1) the moment-preserving threshold algorithm of (Tsai, 1985), which was used for segmentation of the restored image; (2) an edge-tracing algorithm to define the boundary (perimeter) of root tissues; (3) an algorithm to remove objects external to the root system from the image; and (4) algorithms to estimate global traits of the root system (total root length, total root cross-sectional area, total root perimeter, convex hull of the root system) on 2D images (Fig. 1c). SmartRoot was used to produce a skeleton of interconnected individual roots that defines RSAs (Lobet *et al.*, 2011). Throughout the text, root traits are referred to as ‘static’ if the measure is obtained at a single timepoint, such as at the end of an experiment, or ‘dynamic’ if the measure integrates multiple timepoints during an experiment. Root traits are termed ‘global’ when derived from an entire root system or ‘local’ when the measurement refers only to a portion of the root system.

### Statistical analysis

The experiment with a single genotype (*B. rapa* subsp. *trilocularis* cv. R-o-18) was used to calculate the number of replicates (R) that would be required to detect a significant difference between two populations with identical standard deviations in a trait using a two-sided 95% confidence interval (CI) t-test if the trait means differed by 50% (Supplementary Data Equation S1 available at *JXB* online; Eng, 2003).

The sources of variation in static root traits in the single-genotype experiment were determined using a mixed-effects model with experimental run and scanner considered as random factors (Supplementary Data Equation S2). The sources of variation in static root traits in the multiple-genotype experiment were determined using a mixed-effects model with experimental run, scanner, and genotype considered as random factors (Supplementary Data Equation S3). The sources of variation in dynamic root traits were determined using mixed-effects models with genotype and DAS considered as random factors. Logistic growth functions were used to model the increase in total root length and primary root length with time (Supplementary Data Equation S4). The growth rate of a lateral root was expressed as the quotient of the lateral root length divided by the length of time after its emergence from the primary root. A quadratic function was used to model the growth rate of lateral roots (Supplementary Data Equation S6).

Statistical analyses of static root traits were performed using GenStat release 14.1 (VSN International, Oxford, UK). Statistical analyses of all mixed-effects models were performed using R software and the nlme library (Pinheiro and Bates, 2000; R Development Core Team, 2012; Pinheiro *et al.*, 2013).

## Results

### A new platform for high-resolution quantification of root architectural development

Plants grew vigorously for up to 15 days in the pouch-and-wick system and showed no symptoms of mineral deficiencies when provided with an appropriate nutrient solution through the wick. Images of roots were acquired daily with no manual intervention. The custom-written ArchiScan software was used to control the acquisition of images by multiple scanners and computers. Using the customized macros, it was possible to measure global RSA traits from these images and detailed architectural parameters of root systems at the end of experiments were extracted using SmartRoot (Lobet *et al.*, 2011). Total root length estimated using the custom-written macros was highly correlated with total root length estimated using SmartRoot (*R* ^2^=0.77, *n*=20; data not shown). However, the macros generally underestimated total root length, probably because they did not detect extremely fine root features.

### Sources of variation in static root traits of a single *B. rapa* genotype

The root system of the genotype studied in detail, *B. rapa* L. subsp. *trilocularis* cv. R-o-18, consisted of a single primary root and several first-order lateral roots which emerged from the primary root. The emergence of second-order laterals was rarely observed and these roots were therefore not included in any analyses. Coefficients of variation (CVs) for root traits ranged from 5.8 for lateral root insertion angle to 83.2 for lateral root length. Most of the variation in all the traits examined, except for lateral root insertion angle and lateral root length, could be attributed to vagaries in experimental conditions (i.e. run and scanner). Using Supplementary Data Equation S1, it can be calculated that between one and 43 replicates, depending upon the trait, would be required to detect a significant difference using a two-sided 95% CI t-test if trait means differed by 50% (Table 1).

**Table 1.** Sources of variation in shoot and root traits assayed at 18 DAS among 72 surviving seedlings of *Brassica rapa* L. subsp. *trilocularis* cv. R-o-18 grown for 15 days in the phenotyping platformThe experiment was performed in five runs employing eight scanners per run and two plants per scanner. mean, mean trait value; CV, coefficient of variation (*n*=72 seedlings); $\sigma_{a}^{2}$, estimated variance associated with the effect of the run; $\sigma_{b}^{2}$ estimated variance associated with the effect of the scanner; σ^2^, estimated variance associated with the residual error; R, number of replicates required to detect a significant difference in a measured trait between two populations with identical standard deviations in the trait using a two-sided 95% confidence interval t-test.

Trait	Trait means, coefficients of variation, and standard deviations of effects					Source of variation (%)			R
	Mean	CV (%)	$\sigma_{\;a}^{\;2}$	$\sigma {\;}_{b}^{2}$	σ^ 2^	Run	Scanner	Residual	
Shoot fresh weight (mg)	116.6	23.1	27.22	18.36	19.22	42.0	28.3	29.7	3.3
Shoot dry weight (mg)	9.1	19.9	0.68	1.20	1.32	21.4	37.5	41.1	2.4
Root fresh weight (mg)	35.4	40.7	4.17	10.91	9.31	17.1	44.7	38.2	10.2
Root dry weight (mg)	3.2	33.5	0.62	0.79	0.70	29.3	37.6	33.1	6.9
Primary root length (cm)	12.0	31.4	3.05	1.83	3.17	37.9	22.7	39.4	6.1
Primary root diameter (mm)	0.49	9.7	0.070	0.000	0.047	60.1	0.0	39.9	0.6
Lateral branching density (cm^–1^)	2.61	36.8	0.451	0.613	0.713	25.4	34.5	40.1	8.3
Lateral root length (cm)	2.90	83.2	1.96	0.00	2.33	45.7	0.0	54.3	42.5
Lateral root diameter (mm)	0.38	7.6	0.080	0.010	0.026	68.6	8.9	22.5	0.4
Lateral root insertion angle (°)	77.3	5.8	1.58	1.45	4.15	22.0	20.2	57.8	0.2
Total lateral root length (cm)	101.3	40.3	29.7	27.6	29.9	34.1	31.7	34.3	10.0
Total root length (cm)	112.0	37.1	29.5	28.4	30.2	33.5	32.2	34.3	8.5

### Genotypic variation in root traits

A significant effect of genotype (*P*<0.001) was observed for all root traits measured on parents and RILs of the BraIRRI population (Table 2). The parental genotypes exhibited extreme values for many biomass and root length traits. The R500 genotype had the largest values for the majority of root and shoot traits assayed. However, although the IMB211 genotype had the smallest values for total lateral root length and total root length, it did not have the lowest values for all root and shoot traits, providing some evidence for transgressive segregation. Neither parental genotype had the most extreme values for lateral branching density, length or diameter of lateral roots, or lateral root insertion angle.

**Table 2.** Genotypic variation in shoot and root traits assayed at 18 DAS among the parents (IMB211, R500) and 14 recombinant inbred lines of the *Brassica rapa* BraIRRI mapping population grown for 15 days in the phenotyping platformA significant effect of genotype was observed for all traits measured (*P*<0.001, *n*=190 plants). LSD=least significant difference.

	IMB 211	R 500	IRRI 002	IRRI 016	IRRI 030	IRRI 070	IRRI 104	IRRI 124	IRRI 143	IRRI 198	IRRI 201	IRRI 205	IRRI 229	IRRI 248	IRRI 360	IRRI 380	LSD
Shoot fresh weight (mg)	29.4	104.1	55.9	56.3	21.3	41.6	44.4	47.1	60.3	74.0	62.6	34.8	36.9	40.9	64.8	75.3	9.63
Shoot dry weight (mg)	2.0	5.8	3.1	3.2	1.4	2.6	2.8	2.7	3.3	3.7	3.2	2.1	2.4	2.6	4.1	4.7	0.63
Root fresh weight (mg)	8.2	22.6	13.0	12.2	6.7	9.2	12.2	11.5	10.0	13.2	12.3	9.3	10.7	10.2	16.1	21.9	4.05
Root dry weight (mg)	0.8	2.0	2.4	1.0	0.6	1.4	0.9	1.1	1.0	1.1	1.0	0.8	0.9	0.8	1.4	1.9	0.8
Primary root length (cm)	13.1	20.4	17.7	11.9	17.2	14.3	13.4	15.9	18.5	19.5	18.8	16.3	15.1	16.4	18.2	17.9	2.04
Primary root diameter (mm)	0.36	0.41	0.37	0.40	0.33	0.36	0.38	0.35	0.35	0.35	0.36	0.35	0.34	0.35	0.38	0.38	0.003
Lateral branching density (cm^–1^)	2.65	3.15	3.23	2.46	1.68	3.43	3.04	2.71	2.79	2.12	2.25	2.79	2.83	2.59	3.07	3.81	0.540
Lateral root length (cm)	1.33	1.35	1.03	1.14	2.02	1.37	1.78	1.10	0.69	0.77	1.03	1.34	1.74	0.76	1.07	1.74	0.487
Lateral root diameter (mm)	0.26	0.26	0.33	0.31	0.23	0.24	0.24	0.27	0.28	0.29	0.26	0.23	0.24	0.25	0.31	0.27	0.036
Lateral root insertion angle (º)	70.1	70.8	72.9	63.3	75.1	66.8	65.9	65.6	73.4	62.8	63.8	68.8	65.1	65.2	63.3	71.0	4.12
Total lateral root length (cm)	15.7	74.7	30.0	22.5	18.5	24.7	33.9	23.8	22.3	22.8	24.4	32.4	39.1	19.4	31.8	57.7	13.01
Total root length (cm)	28.8	95.0	47.7	34.4	35.8	38.9	47.3	39.7	40.7	42.2	43.2	48.4	54.2	36.3	52.1	75.6	13.47

**Fig. 2.**
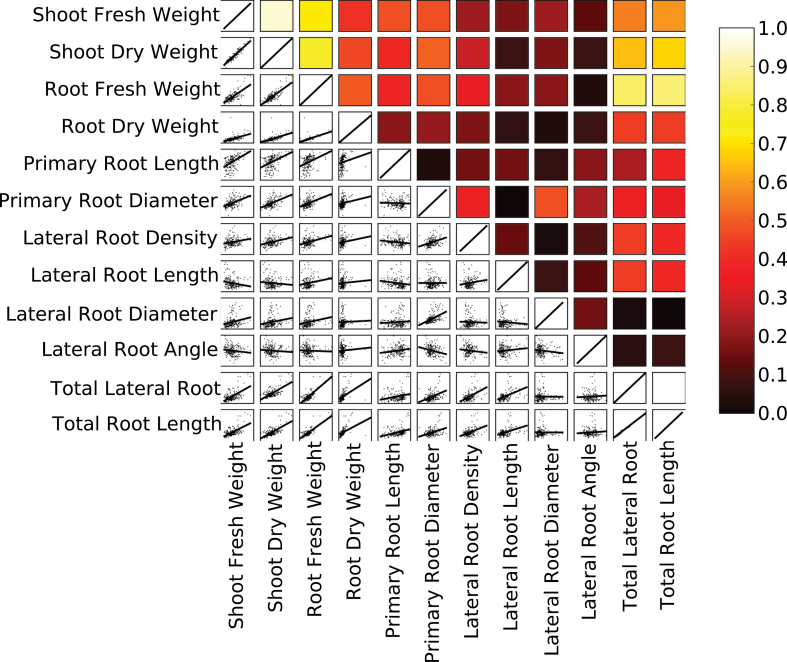
Correlations between plant traits. The plots below the diagonal show linear regressions (red lines) of the data (black points) for different traits. The correlation coefficients for these linear regressions are indicated by the colour of the boxes above the diagonal. The scale of colour codes indicates the correlation coefficients between two traits.

There were strong positive correlations among biomass traits among the 190 plants studied (Fig. 2). Total root length was strongly positively correlated with shoot and root biomass, total lateral root length, lateral root branching density on the primary root, and lateral root length. The diameter of the primary root was also correlated with shoot and root biomass and with the diameter of lateral roots. Little correlation was found between either primary root length or lateral root insertion angle and any other trait.

Little variation in the traits assayed was attributed directly to run or scanner in the multiple-genotype experiment. The effects of genotype, and the effects of interactions between genotype×run, genotype×scanner, and genotype×run×scanner accounted for most of the experimental variation (Table 3). The effect of genotype alone accounted for more variation in shoot traits than in root traits. The effect of genotype alone accounted for >44% of the variation in shoot biomass, but only 15–23% of the variation in root biomass. Broad-sense heritability, estimated as the quotient of the estimated variance associated with the genotypic effect and the total variance for the trait ($\sigma_{g}^{2}$/$\sigma_{p}^{2}$), was largest for shoot biomass traits (>0.60), intermediate (0.25–0.60) for length and diameter of primary roots and lateral root branching density on the primary root, and smallest (<0.25) for root biomass traits, lateral root length, lateral root diameter, lateral root insertion angle, total lateral root length, and total root length (Table 3).

**Table 3.** Sources of variation and broad-sense heritability in shoot and root traits assayed at 18 DAS among 190 surviving seedlings of the parents (IMB211, R500) and 14 recombinant inbred lines of the *Brassica rapa* BraIRRI mapping population grown for 15 days in the phenotyping platformm=mean trait value; $\sigma {\;}_{g}^{2}$, estimated variance associated with the effect of genotype; $\sigma {\;}_{ag}^{2}$, estimated covariance associated with the effect of genotype×experimental run; $\sigma {\;}_{bg}^{2}$, estimated covariance associated with the effect of genotype×scanner; $\sigma {\;}_{abg}^{2}$, estimated covariance associated with the effect of genotype×experimental run×scanner; $\sigma {\;}_{bg}^{2}$, estimated variance associated with the residual error, *H* ^*2*^, broad-sense heritability.

Trait	Trait means and standard deviations of effects							Source of variation (%)				
	Mean	$\sigma_{g}$	$\sigma_{ag}$	$\sigma_{bg}$	$\sigma_{abg}$	σ	*H* ^*2*^	Genotype	Genotype× Run	Genotype× Scanner	Genotype× Run×Scanner	Residual
Shoot fresh weight (mg)	53.4	19.58	9.17	1.38	0.00	10.10	0.72	48.7	22.8	3.4	0.0	25.1
Shoot dry weight (mg)	3.1	1.02	0.66	0.00	0.00	0.61	0.60	44.4	28.9	0.0	0.0	26.7
Root fresh weight (mg)	12.5	2.94	4.44	0.00	2.30	3.32	0.21	22.6	34.1	0.0	17.7	25.6
Root dry weight (mg)	1.2	0.32	0.26	0.00	0.88	0.56	0.12	15.7	13.0	0.0	43.5	27.7
Primary root length (cm)	16.4	2.19	1.18	0.00	1.24	2.34	0.41	31.4	17.0	0.0	17.8	33.7
Primary root diameter (mm)	0.36	0.019	0.014	0.007	0.011	0.019	0.36	27.8	19.6	9.5	15.4	27.7
Lateral branching density (cm^–1^)	2.82	0.454	0.322	0.155	0.000	0.666	0.28	28.4	20.1	9.7	0.0	41.7
Lateral root length (cm)	1.28	0.16	0.44	0.14	0.16	0.48	0.06	11.9	31.7	10.1	11.6	34.7
Lateral root diameter (mm)	0.27	0.000	0.040	0.007	0.022	0.023	0.00	0.0	43.6	7.6	24.0	24.8
Lateral root insertion angle (°)	67.6	3.12	3.48	1.62	0.36	4.18	0.24	24.4	27.3	12.7	2.8	32.7
Total lateral root length (cm)	31.3	8.09	18.35	1.99	1.25	9.15	0.14	20.8	47.3	5.1	3.2	23.6
Total root length (cm)	47.8	9.73	18.75	1.23	2.15	9.45	0.18	23.6	45.4	3.0	5.2	22.9

### The dynamics of root growth

**Fig. 3.**
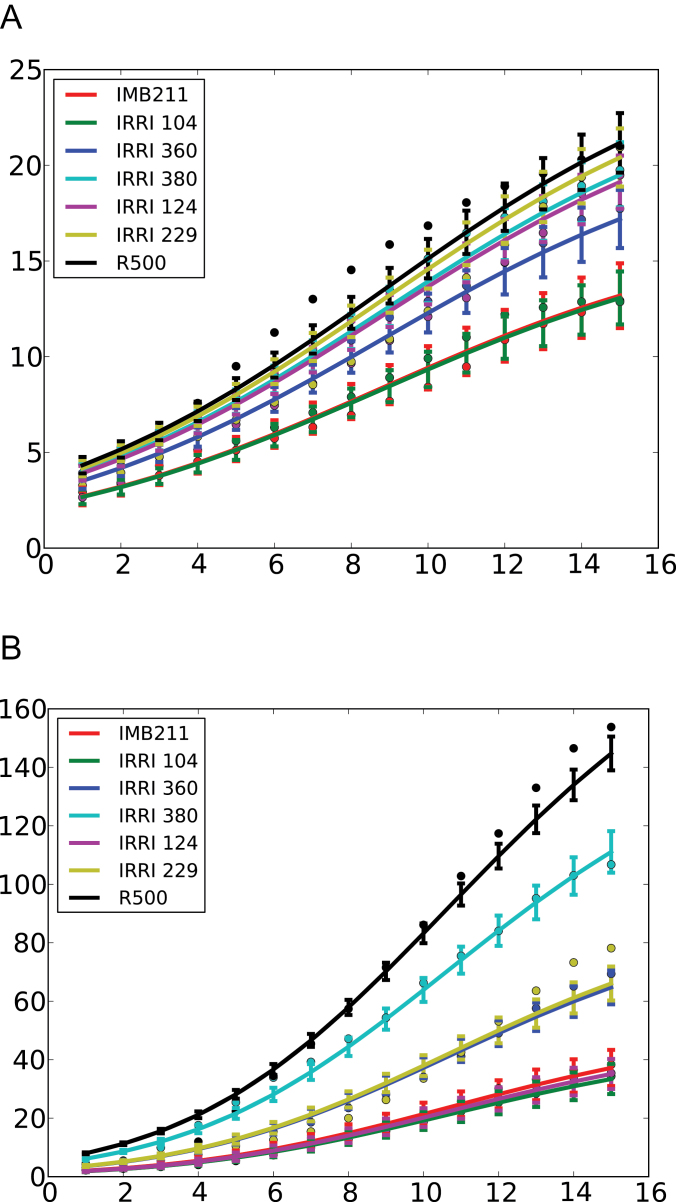
Measured (circles) and predicted (lines) values of primary root length (A) and total root length (B) of the two parents and five recombinant inbred lines of the *Brassica rapa* BraIRRI mapping population over the 15 d following transfer to the phenotyping platform. Predicted values were estimated using a nonlinear mixed-effects model (Supplementary Data Equation S4). Error bars indicate standard error of the predicted means.

Primary root length and total root length were measured daily during the course of the experiment (Fig. 3). The increases in primary root length and total root length with time followed a sigmoidal shape for all genotypes and the data showed no irregularities. The model that fitted the combined data for all genotypes best was a logistic growth function. The most informative model included only a single, random-effect parameter (the asymptote, ${Ø}_{1}$, Supplementary Data Equation S4) describing the effect of genotype on the growth in primary root length or total root length. Both the inflection point (${Ø}_{2}$) and scale parameter of the logistic growth function (${Ø}_{3}$) were constants across all genotypes studied. Values for the inflection point and scale parameter of the logistic growth function describing primary root length were 8.82 DAS and 0.211, respectively. Values for the inflection point and scale parameter of the logistic growth function describing total root length were 10.4 DAS and 0.310, respectively. Asymptotes for primary root length and total root length differed between genotypes (Table 4). The parental genotype IMB211 had an asymptote of 17.4cm and the parental genotype R500 had an asymptote of 28.3cm for primary root length. The parental genotype IMB211 had the smallest asymptote (37.3cm) and the parental genotype R500 had the largest asymptote (126.6cm) of all the genotypes assayed for total root length. These observations are consistent with the measurements of primary root length and total root length assayed at 18 DAS (Table 2).

**Table 4.** Estimates of the asymptotes (${Ø}_{2}$, Supplementary Data Equation S4) for mixed-effects models describing temporal variation in total root length and primary root length, and the intercept ($b_{il}+\beta_{1}$, Supplementary Data Equation S6) for the mixed-effects model describing the growth rate of first-order lateral roots among the parents (IMB211, R500) and 14 recombinant inbred lines of the *Brassica rapa* BraIRRI mapping population grown for 15 days in the phenotyping platform

	IMB 211	R 500	IRRI 002	IRRI 016	IRRI 030	IRRI 070	IRRI 104	IRRI 124	IRRI 143	IRRI 198	IRRI 201	IRRI 205	IRRI 229	IRRI 248	IRRI 360	IRRI 380
Total root length	37.3	126.6	64.2	49.4	47.8	56.8	55.9	47.5	61.1	56.1	53.5	61.3	67.6	52.6	71.5	113.7
Primary root length	17.4	28.3	22.3	17.0	22.9	19.1	17.1	20.3	25.4	29.0	26.5	21.1	22.5	23.5	19.1	24.0
Lateral growth rate	0.255	0.290	0.255	0.273	0.307	0.285	0.326	0.262	0.216	0.234	0.263	0.296	0.295	0.233	0.281	0.297

**Fig. 4.**
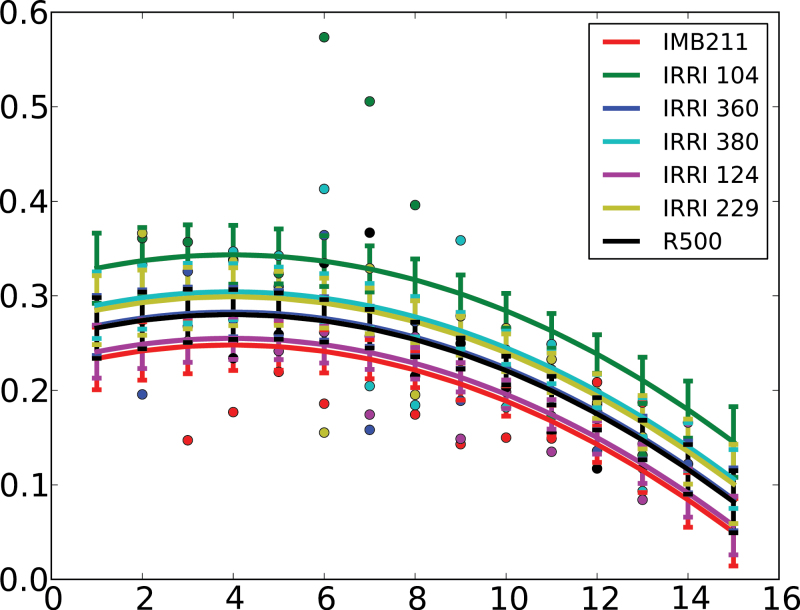
Measured (circles) and predicted (lines) values of the elongation rates of lateral roots of the two parents and five recombinant inbred lines of the *Brassica rapa* BraIRRI mapping population as a function of the time of their emergence after transfer to the phenotyping platform. Predicted values were estimated using a nonlinear mixed-effects model (Supplementary Data Equation S6). Error bars indicate standard error of the predicted means.

The relationship between the growth rate of first-order lateral roots and their day of emergence after transfer to the phenotyping platform followed a quadratic function for all genotypes (Fig. 4). The lateral roots that emerged first (the basal lateral roots) generally had faster elongation rates than those that emerged later. The maximum lateral root elongation rate predicted by the model fitted to the data was 0.35cm d^–1^. The most informative model included only one, random-effect parameter (*b*_i1_, Supplementary Data Equation S6) describing the effect of genotype on the initial growth rate of first-order lateral roots. The maximum initial growth rate (*b*_i1_ + *β*_1_) of first-order lateral roots ranged from 0.216 to 0.307cm d^–1^, with IMB211 having a value of 0.255cm d^–1^ and R500 having a value of 0.290cm d^–1^. Unexplained residual errors in the model for lateral root elongation rate (Fig. 4) were greater than those for the models for either primary root length or total root length (Fig. 3).

## Discussion

### Screening for root traits that improve crop yield

The efficient acquisition of a particular mineral element requires a specific set of root traits, many of which have been identified (Dunbabin *et al.*, 2003; Lynch, 2007, 2013; White *et al.*, 2013a,b). However, breeding for such root traits, either directly through screening for the traits themselves or indirectly through the identification of molecular-markers associated with the traits, requires observations on large populations of genotypes, which necessitates high-throughput, low-cost phenotyping platforms. Imaging is the central component of high-throughput phenotyping. Images contain detailed quantitative information on plant shape and morphologies. Images can be acquired rapidly and without manual intervention using, for example, motorized positioners and conveyors to move samples and/or image capture equipment (Yazdanbakhsh and Fisahn, 2009; Hartmann *et al.*, 2011). Image data can be obtained at regular time intervals for a large number of samples. Image analysis algorithms can then be used to extract biologically meaningful traits automatically from image data (Armengaud *et al.*, 2009; Lobet *et al.*, 2011). Unfortunately, the development of such systems requires considerable expertise in engineering and computer programming and large monetary investments in infrastructure. These prerequisites are often lacking in plant research laboratories. Root images are also noisier that shoot images and the image resolution obtained with conventional cameras can be a limitation to phenotyping.

The phenotyping platform developed for the experiments reported here can overcome some of these limitations. The platform requires no sophisticated equipment and is adaptable to most laboratory and glasshouse environments. The utility of flatbed scanners for high-resolution imaging of roots was recently demonstrated by Dresbøll *et al.* (2013) in their study of the responses of tomato root growth to anoxia. The quality of the images obtained when roots were imaged against the uniform background of the germination paper using the scanners enabled efficient image analysis (Fig. 1). The ability to control multiple scanners automatically allowed the acquisition of images over extended periods without manual intervention and will enable the extension of phenotyping platform to the simultaneous screening of larger populations of genotypes.

### Variation in root growth among *B. rapa* genotypes

Coefficients of variation for specific shoot and root traits measured in 72 individuals of R-o-18 varied considerably (Table 1). The relationship between the CV for a trait and the number of replicates required to detect a significant difference follows a quadratic function. Since the CVs for many root traits were large, many replicates would be required to detect differences in these traits between genotypes. Since plants were grown on the surface of a germination paper with homogeneous distribution of water and mineral nutrients, most of the variability observed in root traits is intrinsic to the processes of root development. Intrinsic noise, or developmental stochasticity, is particularly significant in plant roots. It has been shown, for example, that innate changes in lateral root growth rates can exceed by up to 5-fold those observed in response to nitrate availability (Forde, 2009). It is essential therefore to develop ways to characterize developmental stochasticity in order to minimize residual variations in root phenotyping experiments.

The phenotyping platform developed here was used to quantify variation in shoot and root morphological traits from a selection of genotypes (Table 2). A significant effect of genotype was observed for all traits. It is usually observed that shoot biomass traits have larger broad-sense heritability that root biomass traits (Arraouadi *et al.*, 2012; Bouteillé *et al.*, 2012), and the results obtained with the pouch-and-wick system confirm these observations (Table 3). Broad-sense heritability of root length typically ranges from 0.15 to 0.80 (Gahoonia and Nielsen, 2004; Bouteillé *et al.*, 2012). Although shoot biomass, primary root length, and lateral root branching density have large CVs, their heritability is high. Traits with large differences between genotypes and small variation within a genotype (i.e. high hereditability) require less replication to detect significant differences between genotypes than traits with either small differences between genotypes or low hereditability.

Many of the static traits described above show temporal variation. For example, primary root length and total root system length follow a sigmoidal growth function with time (Fig. 3). This has been observed in many crop species (Merrill *et al.*, 2002). It is also common to observe fastest growth rates of lateral roots emerging a few days after sowing (Nacry *et al.*, 2005). This behaviour was best modelled on the current data using a quadratic relationship between lateral root growth rate and day of emergence from the primary root (Fig. 4). However, lateral root growth rate within a single plant was highly variable and the residual variances in models were higher than in primary and total root length models. Although run was not a significant factor, there were large differences in the variance of the residual between two runs for the total root length models, and different models might be required in the future for the analysis of such data. There were significant effects of genotype on the dynamics of root growth (Table 4 and Figs 3 and 4). Data for primary root length and total root length indicate that all genotypes follow a similar growth pattern with time but differ in their absolute growth rate. Data for the elongation rate of lateral roots also indicate that all genotypes follow a similar pattern with time, but differ in their maximum growth rate.

### The application of scanner-based high-resolution root phenotyping

Identifying chromosomal loci affecting a particular root trait (quantitative trait loci) requires evaluation of many hundreds of genotypes with high replication (Qu *et al.*, 2008; Sharma *et al.*, 2011; Shi *et al.*, 2013). The phenotyping platform described here can automatically image the root systems of 48 plants grown simultaneously (Fig. 1). It is, therefore, not of a sufficient size to phenotype the root systems of a genetic mapping population within a short time period. However, since it has no moving parts, it is relatively simple to extend the platform, although achieving the required throughput might necessitate a reduction in the cost of the equipment. This could be achieved for example with a more economical imaging technology such as contact image sensors (Dannoura *et al.*, 2012).

Real soil environments are also difficult to reproduce in laboratories and glasshouses. Roots grow in three dimensions and experience a range of physical conditions that influence their growth in various ways (Bengough *et al.*, 2011). Interactions with a range of biological organisms, such as bacteria and arbuscular mycorrhizal fungi, can also have a strong impact on the acquisition of water and mineral elements (Bucher, 2007). The ability to image root systems of mature plants growing in soil is likely to improve the correlations between traits obtained in the phenotyping platform and measurements made in the field. The platform described here can be used to image the roots of plants growing in other substrates, including soil (Dresbøll *et al.*, 2013). Furthermore, although the platform described here could only accommodate young seedlings because of the size of the scanner window, root systems of larger plants might be accommodated by growing plants in larger pouches, which could be imaged in overlapping sectors and these images combined to reconstruct an image of the entire root system, as described recently by Lobet and Draye (2013).

Root systems of plants grown in soil respond dynamically to changes in their local environment. For example, the development of RSAs alters in response to vagaries in the availability of water (Taylor and Ratliff, 1969; Rostamza *et al.*, 2013), the root distribution within the soil profile responds to the presence of macropores and the depth at which a compacted subsoil is formed (Valentine *et al.*, 2012; Acuña and Wade, 2013), lateral roots proliferate in patches of soil with high availability of various essential mineral elements (Forde and Walch-Liu, 2009; Hodge *et al.*, 2009), and root systems develop to avoid exposure to toxic elements, such as cadmium (Lux *et al.*, 2011). The platform described here can be used to investigate responses of the root systems to environmental variables and, if scaled to accommodate genetic mapping populations, to identify genetic factors affecting root responses to the environment. The ability to characterize dynamic responses to environmental variables will allow researchers to identify quantitative trait loci influencing the plasticity of the root system, which is required for marker-assisted selection of genotypes adapted to multiple soil types and environmental conditions (de Dorlodot *et al.*, 2007; Hochholdinger and Tuberosa, 2009; Hodge *et al.*, 2009; Acuña and Wade, 2013). The application of scanner-based, high-resolution root phenotyping of mature plants grown in soil could, therefore, facilitate the development of crop varieties that are better adapted to future environmental conditions.

## Supplementary material

Supplementary data are available at *JXB* online.

Supplementary Data. Statistical models of root systems.

Supplementary Data
