# Supplementary material for: A scanner system for high-resolution quantification of variation in root growth dynamics of Brassica rapa genotypes
Source: J Exp Bot. 2014 Mar 6;65(8):2039–48. doi: 10.1093/jxb/eru048 (PMC3991737; doi:10.1093/jxb/eru048)
Supplement: Supplementary Data [file supp_65_8_2039__index.html]

A scanner system for high-resolution quantification of variation in root growth dynamics of Brassica rapa genotypes — A scanner system for high-resolution quantification of variation in root growth dynamics of Brassica rapa genotypes — Supplementary Data 

# A scanner system for high-resolution quantification of variation in root growth dynamics of *Brassica rapa* genotypes

## Supplementary Data

Data files

**Files in this Data Supplement:**

- Supplementary Data - Supplementary Data
- Supplementary Data - Supplementary Data
