# Supplementary material for: A scanner system for high-resolution quantification of variation in root growth dynamics of Brassica rapa genotypes
Source: J Exp Bot. 2014 Mar 6;65(8):2039–48. doi: 10.1093/jxb/eru048 (PMC3991737; doi:10.1093/jxb/eru048)
Supplement: Supplementary Data [file supp_eru048_jexbot109439_file002.pdf]

High-resolution quantification of variation in root growth dynamics of *Brassica rapa* genotypes. *Michael O Adu, Antoine Chatot, Lea Wiesel, Malcolm J Bennett, Martin R Broadley, Philip J White, and Lionel X Dupuy*

SUPPLEMENTARY DATA

Statistical models of root systems

The experiment with a single genotype (*B. rapa* subsp. *trilocularis* cv. R-o-18) was used to calculate the number of replicates (*R*) that would be required to detect a significant difference between two populations with identical standard deviations in a trait using a two-sided, 95% confidence interval (CI), t-test, if the trait means differed by 50% (Eng, 2003):

$$R = \left(\frac{CV\ Z_{crit}}{25}\right)^2, \qquad \text{(S1)}$$

where *CV* is coefficient of variation for the trait in the reference population and Z_{crit} is the standard normal deviate corresponding to a confidence interval of 95% (1.96).

The sources of variation in static root traits in the single-genotype experiment were determined using a mixed effects model with experimental run and scanner considered as random factors:

$$y_{ij} = m + a_i + b_j + \epsilon_{ij},$$
$$i \in \{1, \dots, n\}, j \in \{1, \dots, r\}, \qquad \text{(S2)}$$
$$a_i \sim N(0, \sigma_a^2), \qquad b_j \sim N(0, \sigma_b^2), \qquad \epsilon_{ij,} \sim N(0, \sigma^2),$$

where y_{ij} represents the root trait from the ith run and j^{th} scanner, *m* is the mean trait value, a_i is the effect of ith run, b_j is the effect of the jth scanner, ϵ_{ij} is the residual error, *n* is the number of runs (5), *r* is the total number of scanners (8), σ_a^2 is the estimated variance associated with the effect of the run, σ_b^2 is the estimated variance associated with the effect of the scanner, and), σ^2 is the estimated variance associated with the residual error.

The sources of variation in static root traits in the multiple-genotype experiment were determined using a mixed effects model with experimental run, scanner and genotype considered as random factors:

$$y_{ijk} = m + g_k + ag_{ik} + bg_{jk} + abg_{ijk} + \epsilon_{ijk}, \qquad \text{(S3)}$$
$$i \in \{1, \dots, n\}, j \in \{1, \dots, r\}, k \in \{1, \dots, s\},$$
$$g_k / ag_{ik} / bg_{jk} / abg_{ijk} \sim N\left(0, \sigma^2_{g/ag/bg/abg}\right), \epsilon_{ij} \sim N(0, \sigma^2),$$

where y_{ijk} represents the root trait from the ith experimental run, j^{th} scanner and k^{th} genotype, m is the mean trait value, g_k is the effect of the genotype, ag_{ik} is the effect of interactions

between experimental run and genotypic factors, bg_{jk} is the effect of interactions between scanner and genotypic factors, abg_{ijk} is the effect of interactions between experimental run, scanner and genotypic factors, ϵ_{ijk} is the residual error, *n* is the number of runs (2), *r* is the total number of scanners (24) and *s* is the number of genotypes (16). Broad-sense heritability (H^2) was estimated as σ_g^2 / σ_p^2, where σ_g^2 is the estimated variance associated with the genotypic effect and σ_p^2 is the total variance for the trait.

The sources of variation in dynamic root traits were determined using mixed effects models with genotype and day after sowing (DAS) considered as random factors. To account for non-linearity in growth curves, a logistic growth function was used to model the increase in total root length and primary root length with time. The three parameters of the logistic function were the asymptote ($\emptyset_1$), inflection point ($\emptyset_2$), and scale parameter ($\emptyset_3$). These models were used to describe sources of temporal variation in (i) total root length, (ii) primary root length, and (iii) the growth rate of first order lateral roots:

$$y_{ij} = \frac{\emptyset_{i1}}{1+\exp\left[-\left(DAS_j - \emptyset_{i2}\right)/\emptyset_{i3}\right]} + \epsilon_{ij}, \qquad \text{(S4)}$$

$$\emptyset_i = \begin{bmatrix}\emptyset_{i1}\\ \emptyset_{i2}\\ \emptyset_{i3}\end{bmatrix} = \begin{bmatrix}\beta_1\\ \beta_2\\ \beta_3\end{bmatrix} + \begin{bmatrix}b_i\\ 0\\ 0\end{bmatrix},$$

$$i = \{1, \dots, s\}, j = \{1, \dots, t\},$$

$$b_i \sim N\left(0, \sigma_b^2\right),\ \epsilon_{ij} \sim N\left(0, \sigma^2\right).$$

where y_{ij} is the total root length or primary root length for the ith genotype, on the jth DAS, and *t* is the number of timepoints at which measurements were made (15). The parameters β_1, β_2 and β_3 are the mean values of the individual logistic parameters $\emptyset_{i1}$, $\emptyset_{i2}$ and $\emptyset_{i3}$, respectively, and b_i, is the random effect on the asymptote of the logistic function and ϵ_{ij} is the residual error. A likelihood ratio test was used to select the final model, which had the three parameters as fixed effects and only the asymptote as a random effect. For the total root length model, data were normalised by square root transformation before analyses. Autocorrelation in the data was modelled using the moving average (corARMA) and autoregressive model of an order 1 (AR1) correlation structure (Pinheiro and Bates, 2000). To account for heteroscedasticity a power variance function was used of the form:

$$Var\left(\epsilon_{ij} \middle| DAS\right) = \sigma^2\left(\delta_1 + |DAS|^{\delta_2}\right), \qquad \text{(S5)}$$

where σ^2 is the variance when *j*=0 and δ_1 and δ_2 are the two parameters for the power variance function (Pinheiro and Bates, 2000).

The growth rate of a lateral root was expressed as the quotient of the lateral root length divided by the length of time after its emergence from the primary root. Data for growth rates of lateral roots were normalized by square root transformation and the sources of variation in the growth rate of lateral roots were determined using mixed effects models with genotype and day after sowing (DAS) considered as random factors:

$$y_{ij} = b_{i1} + \beta_1 + \beta_2 DAS_j + \beta_3 DAS_j^2 + \epsilon_{ij}\ , \qquad \text{(S6)}$$

$$i = \{1, \dots, s\}, j = \{1, \dots, t\},$$

$$b_{i1} \sim N(0, \sigma_{b1}^2), \epsilon_{ijk} \sim N(0, \sigma^2).$$

where y_{ij} is the lateral root growth rate for genotype i on the jth day of the experiment, β_1, β_2 and β_3 are the fixed effect parameters for the quadratic function and b_{i1} is the random effects on the intercept of the quadratic function.

The sources of variation in the mixed effects models described in Equations 2, 3, 5, 7, and 8 were chosen based on Akaike and Bayesain information criteria (Pinheiro and Bates, 2000). The quality of the mixed effects models was also assessed visually using quantile-quantile plots to check for normality and residual plots to check that the variance of residuals was constant (Pinheiro and Bates, 2000). ANOVA was used to determine the significance of differences in fixed and random effects.

References

Eng J. 2003. Sample size estimation: how many individuals should be studied? *Radiology* **227**, 309 -313.

Pinheiro JC, Bates DM. 2000. *Mixed-Effects Models in S and S-PLUS*. New York: Springer-Verlag.
