# Supplementary figures and images for: A scanner system for high-resolution quantification of variation in root growth dynamics of Brassica rapa genotypes
Source: J Exp Bot. 2014 Mar 6;65(8):2039–48. doi: 10.1093/jxb/eru048 (PMC3991737; doi:10.1093/jxb/eru048)

## Slide 1
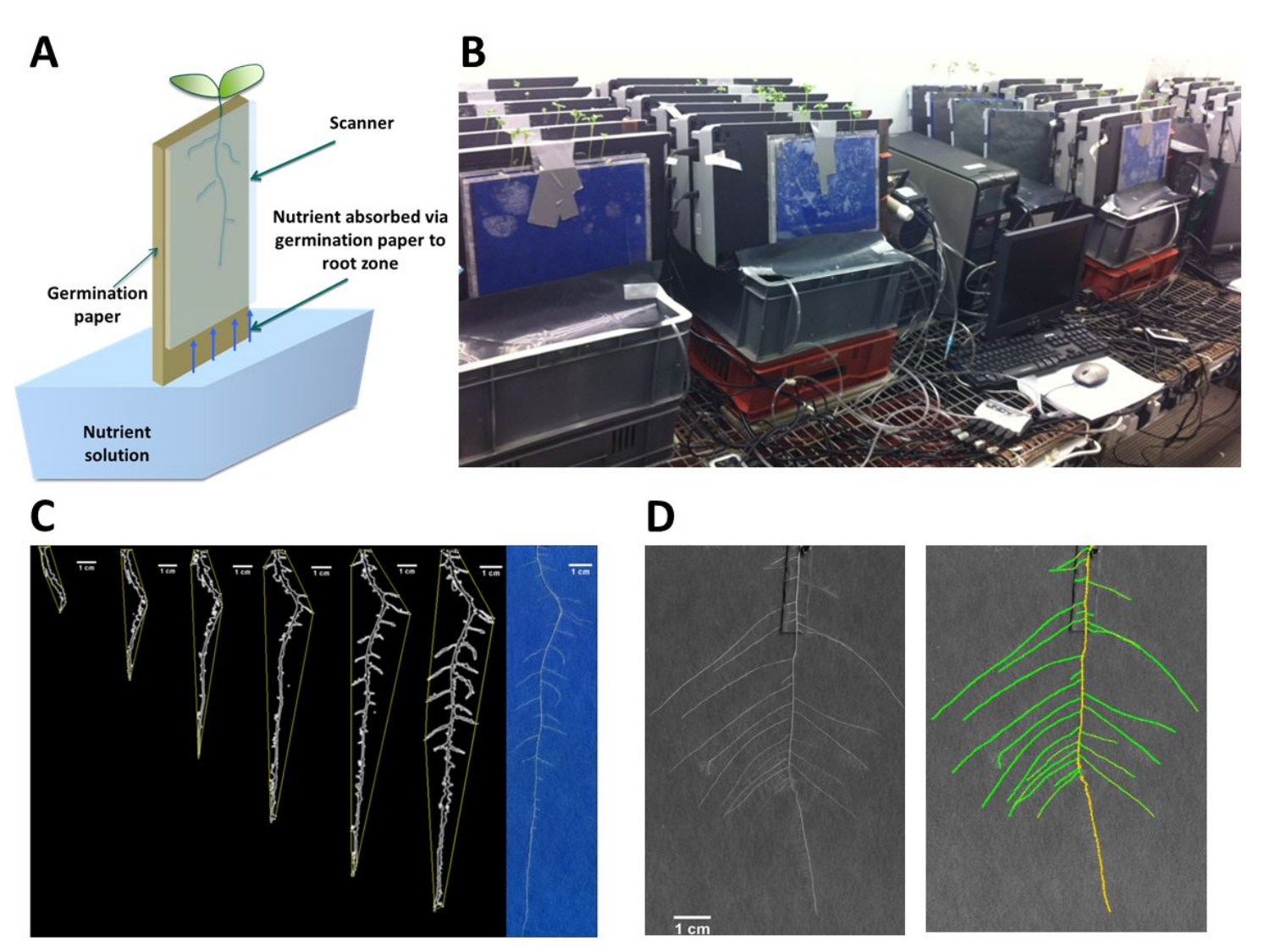

## Slide 2
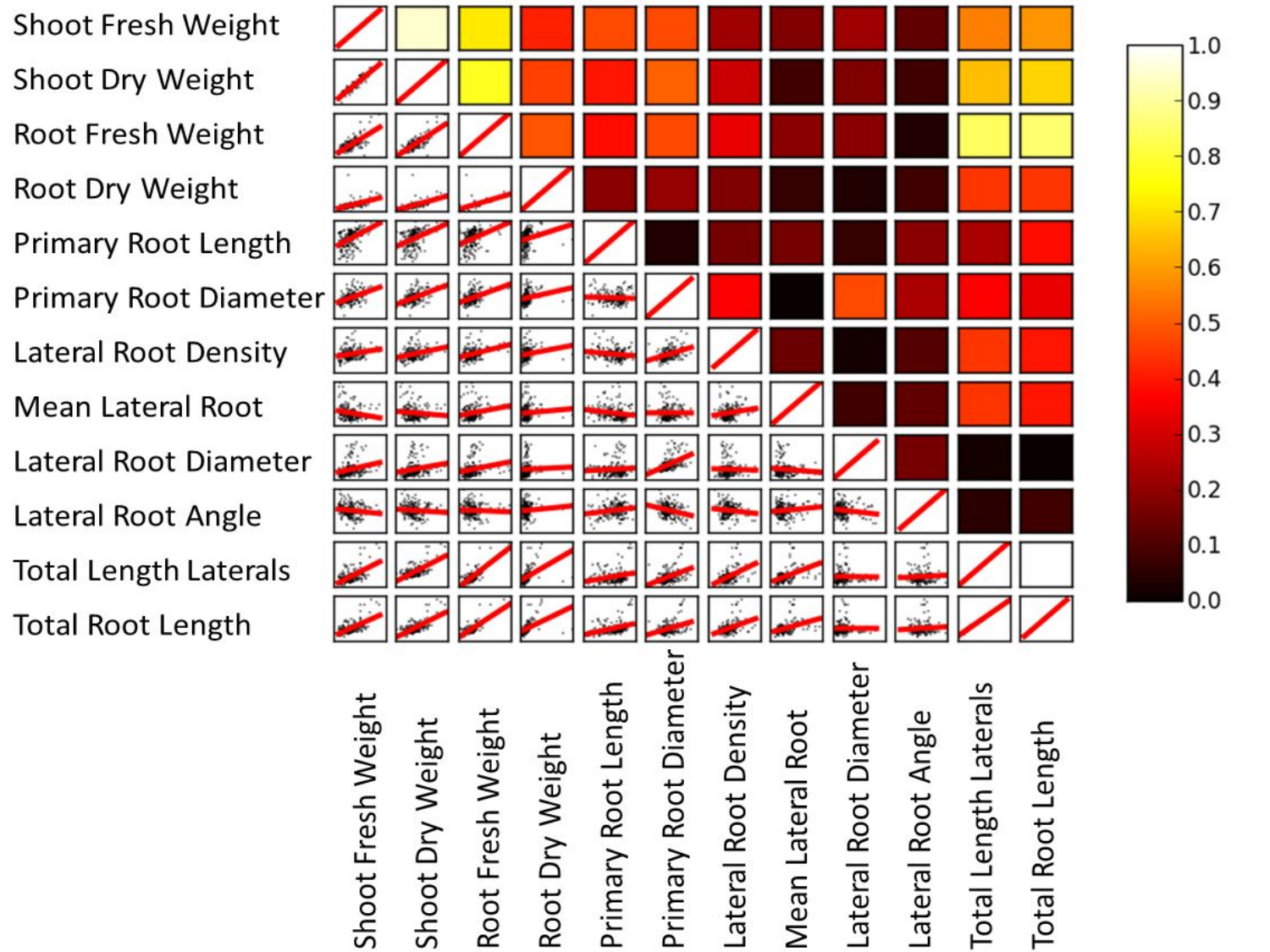

## Slide 3
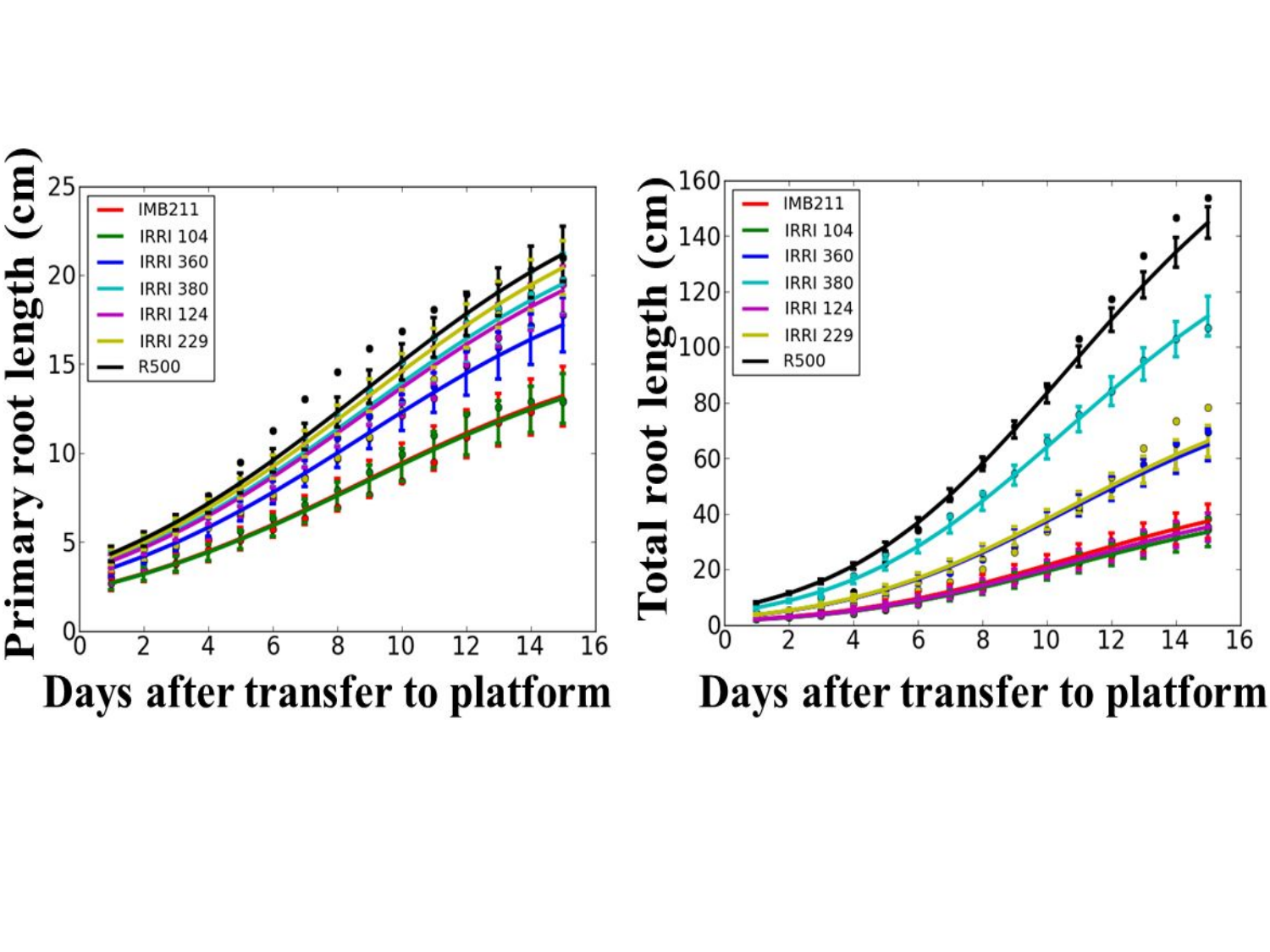

## Slide 4
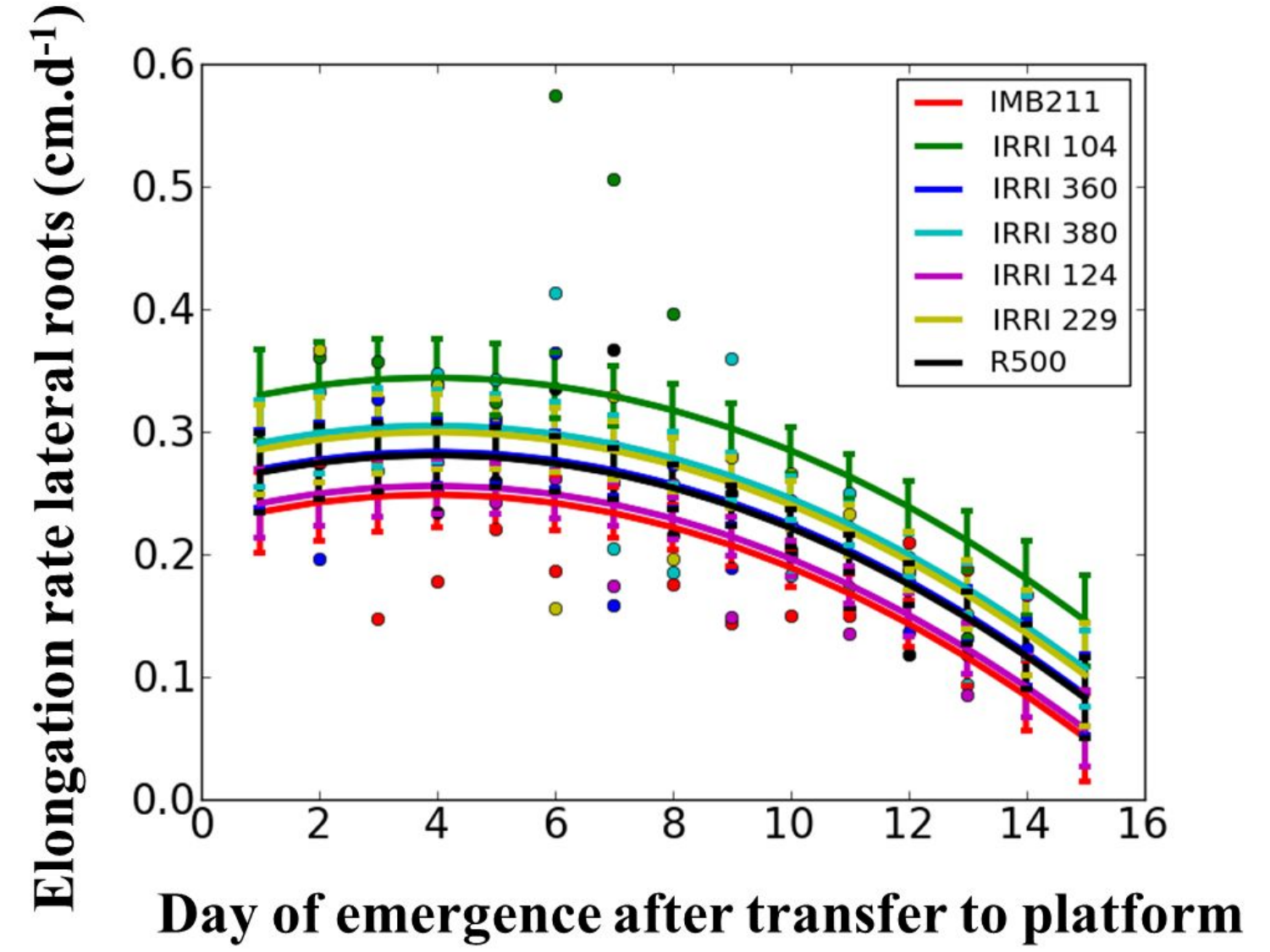

Supplement: Supplementary Data [file supp_eru048_jexbot109439_file001.pptx]
